# Functional outcomes in Hirschsprung disease patients after transabdominal Soave and Duhamel procedures

**DOI:** 10.1186/s12876-018-0783-1

**Published:** 2018-04-27

**Authors:** Amira Widyasari, Winona Alda Pravitasari, Andi Dwihantoro, Gunadi

**Affiliations:** Pediatric Surgery Division, Department of Surgery, Faculty of Medicine, Universitas Gadjah Mada/Dr. Sardjito Hospital, Jl. Kesehatan No. 1, Yogyakarta, 55281 Indonesia

**Keywords:** Constipation, Functional outcomes, Hirschsprung, Pull-through, Soiling, Voluntary bowel movement

## Abstract

**Background:**

Several pull-through procedures have been described for Hirschsprung disease (HSCR) with varying functional outcomes. The voluntary bowel movement (VBM) and the absence of soiling or constipation after pull-through remain the most important markers of good outcome. We aimed to compare the functional outcomes in HSCR patients following Soave and Duhamel procedures.

**Methods:**

Krickenbeck classification was utilized to determine VBM, soiling and constipation for patients who underwent Soave and Duhamel pull-through at Dr. Sardjito Hospital, Indonesia from 2013 to 2016.

**Results:**

Fifty-three patients were ascertained (Soave: 23 males and 2 females vs. Duhamel: 22 males and 6 females, *p* = 0.26). Ninety-three and 88% patients had a VBM following Duhamel and Soave pull-through, respectively (*p* = 0.66). Constipation frequency was significantly higher in Soave than Duhamel groups (24% vs. 4%; *p* = 0.04) with OR of 8.5 (95% CI = 1.0–76.7), whereas soiling rate was similar between Duhamel (21%) and Soave (8%) groups (*p* = 0.26). Furthermore, the risk of constipation was increased ~ 21.7-fold in female patients after Soave procedure and was almost statistically significant (*p* = 0.05).

**Conclusions:**

The constipation rate is higher in patients who underwent Soave than Duhamel procedure, but the VBM and soiling frequencies are similar. The constipation risk following Soave pull-through might be increased by the female gender. Furthermore, a multicenter study with a larger sample of patients is necessary to clarify and confirm our findings.

## Background

Hirschsprung disease (HSCR) is caused by the migration failure of neural crest cells during intestinal development, resulting in an aganglionic colon and causing a functional obstruction in children [1, 2]. According to the length of aganglionosis, HSCR can be classified as follows: 1) short-segment, 2) long-segment, and 3) total colonic aganglionosis (TCA), with a male-to-female ratio of approximately 4:1 [1].

The goal of HSCR treatment is surgical resection of the aganglionic bowel and pulling the ganglionated bowel through to a point just above the dentate line [3]. Several pull-through procedures have been described for Hirschsprung disease (HSCR) with varying functional outcomes [4–8]. There is currently some debate over which pull-through technique offers the best outcome [9]. The voluntary bowel movement (VBM) and the absence of soiling or constipation following pull-through remain the most important markers of good outcome [9]. This study aimed to compare the functional outcomes including VBM, soiling and constipation in HSCR patients following the Soave and Duhamel procedures.

## Methods

### Patients

A retrospective study was conducted with children < 18 year of age with HSCR at Dr. Sardjito Hospital, a University Teaching Hospital [10] in Yogyakarta, Indonesia from January 2012 to December 2016. Fifty-three patients were ascertained (Soave: 23 males and 2 females vs. Duhamel: 22 males and 6 females, *p* = 0.26), corresponding to a sex ratio of 5.6:1 (Table 1).

**Table 1:** Clinical characteristics of HSCR patients who underwent Soave and Duhamel procedures

Characteristics	Soave n (%)	Duhamel n (%)	*p*-value
Gender			
▪ Male	23 (92)	22 (79)	0.26
▪ Female	2 (8)	6 (21)	
Aganglionosis type			0.67
▪ Short-segment	23 (92)	24 (86)	
▪ Long-segment	2 (8)	4 (14)	
Age of HSCR diagnosis	24 mo (0.5–218 mo)	22.5 mo (1–163 mo)	0.93
Age of pull-through			0.66
▪ ≥3 years old	11 (56)	14 (50)	
▪ < 3 years old	14 (44)	14 (50)	
Length of follow-up	17.0 ± 14.3 mo	15.5 ± 14.6 mo	0.71
Nutritional status			0.38
▪ Undernourished	18 (72)	23 (82)	
▪ Well-nourished	7 (28)	5 (18)	

*mo* months, *HSCR* Hirschsprung diseases

Diagnosis of HSCR in our hospital was established according to the clinical manifestation, contrast enema, and histopathology findings. The pathologist utilized the hematoxylin and eosin staining and/or S100 immunohistochemistry for the histopathology diagnosis of HSCR [11–16].

The two-staged Soave and colonic Duhamel pull-through were conducted at our hospital based on our previous study [15]. The definitive surgical procedures were performed by two experienced pediatric surgeons in our institution, and one surgeon only performed one of the techniques. All HSCR patients showed the level of the aganglionic zone at the sigmoid colon, except with six patients which was at the descending colon. Furthermore, there were no TCA patients in our study.

We defined the age of pull-through with the following criteria: ≥3 years old and < 3 years old since surgery in older children presents specific perioperative challenges that might impact the outcomes [17].

In addition, we classified the nutritional status of HSCR patients as follows: undernourished and well-nourished since the peri-operative malnutrition was associated with the functional outcomes following pull-through [3]. Undernourished was defined as weight-for-age Z score < − 2 [18].

This study was approved by the Institutional Review Board of the Faculty of Medicine, Public Health and Nursing, Universitas Gadjah Mada/Dr. Sardjito Hospital, Yogyakarta, Indonesia (KE/FK/1356/EC/2015). Written informed consent was obtained from all parents for participating this study.

### Functional outcomes

Krickenbeck classification was used to evaluate the functional outcomes, including VBM, soiling and constipation, according to previous studies [19–21]. VBM was determined as feeling an urge to defecate, the capacity to verbalize this feeling, and the ability to hold the bowel movement. Soiling was classified into 3 grades as follows: a) grade 1, occasionally soiling (up to once or twice per week); b) grade 2, soiling every day but no social problems; and c) grade 3, constant soiling with social problems, whereas constipation consists of 3 grades: a) grade 1, manageable by changes in diet; b) grade 2, requires laxatives; and c) grade 3, resistant to laxatives and diet [19]. The functional outcomes were assessed by the pediatric surgeons in children ≥3 years old since toilet training is expected by this age [9].

To further investigate the functional outcomes in our HSCR patients after pull-through, we performed a contrast enema to visualize the anatomy after the initial surgery and give insight into the colon motility and any structural defect, or if necessary, a rectal biopsy to rule out a transition zone pull-through or retained aganglionosis, as suggested by previous study [22].

### Statistical analysis

Data were presented as number/percentages and median/mean for categorical and continuous variables, respectively. The Fischer exact, chi-square, Mann-Whitney U, and t tests were used to evaluate the differences between groups.

## Results

We used ICD-10 codes (International Statistical Classification of Diseases and Related Health Problems, 10th Revision) (Q43.1: Hirschsprung disease) to identify patients diagnosed with HSCR and examined 65 medical records. We excluded 12 subjects due to incomplete medical records, thus, we further analyzed 53 infants.

Fifty-three HSCR patients (Soave = 25 vs. Duhamel = 28) had complete data for final analysis (Table 1). Most patients were having short-segment HSCR (89%) and were undernourished (77%). None of the clinical characteristics of HSCR patients showed any difference between the two surgical methods (Table 1).

The VBM rates were 93% and 88% in the Duhamel and Soave groups, respectively, but the differences did not reach a significant level (*p* = 0.66) (Table 2). The soiling frequency was not statistically significant between the Duhamel and the Soave groups (21% vs. 8%, *p* = 0.26) (Tables 2 and 4). In contrast, the constipation rate was significantly higher in the Soave than the Duhamel groups (24% vs. 4%, *p* = 0.04) with OR of 8.5 (95% CI = 1.0–76.7) (Tables 2 and 5). Furthermore, four patients underwent a contrast enema and showed a dilated (hypomotile) colon, a nondilated (hypermotile) colon and an anatomic stricture in one, two and one patients, respectively, while none of the patients underwent a rectal biopsy.

**Table 2:** Functional outcomes in HSCR patients after Soave and Duhamel procedures according to Krickenbeck classification

Functional outcomes	Soave (n, %)	Duhamel (n, %)	*p*-value
Voluntary bowel movements	22/25 (88)	26/28 (93)	0.66
Soiling			
√ Grade 1	2/25 (8)	5/28 (18)	0.26
√ Grade 2	0	1/28 (3)	
√ Grade 3	0	0	
Constipation			
√ Grade 1	1/25 (4)	1/28 (4)	0.04*
√ Grade 2	5/25 (20)	0	
√ Grade 3	0	0	

*, significant (*p* < 0.05)

Next, we analyzed the impact of the gender, aganglionosis type, nutritional status, and age at pull-through on the VBM, soiling and constipation (Tables 3, 4 and 5, respectively). None of the factors affected the functional outcomes after pull-through, but an almost significant effect was observed in the Soave group: the female patients had ~ 21.7-fold higher risk to have constipation after surgery than the male patients (*p* = 0.05) (Table 5).

**Table 3:** Voluntary bowel movements in HSCR patients after Soave and Duhamel pull-through

	Duhamel (n, %)	Soave (n, %)	*p*-value		OR (95% CI)	
			Duhamel	Soave	Duhamel	Soave
Voluntary bowel movements	26/28 (93)	22/25 (88)	0.66		1.7 (0.3–11.1)	
Gender						
√ Male	20/26 (77)	20/22 (91)	0.78	0.92	0.6 (0.03–14.9)	1.2 (0.05–29.9)
√ Female	6/26 (23)	2/22 (9)				
Aganglionosis type						
√ Long-segment	3/26 (11.5)	2/22 (9)	0.19	0.92	0.1 (0.01–2.7)	0.9 (0.03–21.8)
√ Short-segment	23/26 (88.5)	20/22 (91)				
Nutritional status						
√ Undernourished	21/26 (81)	17/22 (77)	0.88	0.15	0.8 (0.03–18.8)	6.8 (0.5–91.5)
√ Well-nourished	5/26 (19)	5/22 (23)				
Age of pull-through						
√ ≥3 years old	14/26 (54)	11/22 (50)	0.27	0.21	5.8 (0.3–132.6)	7.0 (0.3–151.4)
√ < 3 years old	12/26 (46)	11/22 (50)

**Table 4:** Soiling frequency in HSCR patients after Soave and Duhamel pull-through

	Duhamel (n, %)	Soave (n, %)	*ph*-value		OR (95% CI)	
			Duhamel	Soave	Duhamel	Soave
Soiling frequency	6/28 (21)	2/25 (8)	0.26		3.1 (0.6–17.2)	
Gender						
√ Male	6/6 (100)	2/2 (100)	0.29	0.75	5.1 (0.3–104.6)	0.6 (0.02–15.9)
√ Female	0	0				
Aganglionosis type						
√ Long-segment	0	0	0.46	0.75	0.3 (0.01–6.7)	1.7 (0.06–46.9)
√ Short-segment	6/6 (100)	2/2 (100)				
Nutritional status						
√ Undernourished	5/6 (83)	2/2 (100)	0.93	0.61	1.1 (0.1–12.3)	2.2 (0.1–53.4)
√ Well-nourished	1/6 (17)	0				
Age of pull-through						
√ ≥3 years old	4/6 (67)	2/2 (100)	0.36	0.21	2.4 (0.4–15.9)	7.6 (0.3–177.2)
√ < 3 years old	2/6 (33)	0

**Table 5:** Constipation rate in HSCR patients after Soave and Duhamel pull-through

	Soave (n, %)	Duhamel (n, %)	*p*-value		OR (95% CI)	
			Soave	Duhamel	Soave	Duhamel
Constipation frequency	6/25 (24)	1/28 (4)	0.04*		8.5 (1.0–76.7)	
Gender						
√ Female	2/6 (33)	1/1 (100)	0.05	0.14	21.7 (0.9–534.1)	12.2 (0.4–344.1)
√ Male	4/6 (67)	0				
Aganglionosis type						
√ Long-segment	0	0	0.70	0.75	0.5 (0.02–12.8)	1.7 (0.1–49.9)
√ Short-segment	6/6 (100)	1/1 (100)				
Nutritional status						
√ Undernourished	5/6 (83)	1/1 (100)	0.49	0.87	2.3 (0.2–24.3)	0.8 (0.03–21.5)
√ Well-nourished	1/6 (17)	0				
Age of pull-through						
√ ≥3 years old	4/6 (67)	0	0.21	0.49	3.4 (0.5–23.8)	0.3 (0.01–8.3)
√ < 3 years old	2/6 (33)	1/1 (100)				

## Discussion

We clearly show that the VBM and soiling frequencies are similar between the Duhamel and Soave groups, but the constipation rate is higher in the Soave than Duhamel groups. The risk of constipation following the Soave procedure is increased ~ 8.5-fold higher than the Duhamel procedure. This finding might be caused by an anastomotic stricture or “rolling down” of the rectal muscular cuff following the Soave procedure [23]. If there was an anatomic stricture identified in our patients after evaluation by a contrast enema, we managed them with serial dilatation. Also, the constipation rates following the Soave and Duhamel procedures in our study were similar (24% vs. 25%) and lower than (4% vs. 25%) previous study [20].

It has been reported that the HSCR patients who underwent Duhamel procedure will have less soiling [22]. The soiling in the Duhamel group might be caused by the “overflow” incontinence secondary to constipation since the Duhamel technique results in less possibility for the anal canal damage [21, 22, 24]. However, our study showed that the soiling frequency was similar between the Duhamel and the Soave groups. Furthermore, our study focused on the development of soiling and constipation following pull-through, while the enterocolitis after surgery in our cohort patients has been previously reported [15]. As for the VBM, its frequency in this series reached ~ 90% HSCR patients after pull-through and was higher than previous study (67%) [20].

Interestingly, in the Soave group, the female patients had 21-fold higher risk to have constipation following the Soave procedure than the male patients. In the general population without any other gastrointestinal disorders, it has been shown that females have higher constipation rate due to hormonal factors [25]. Unfortunately, we do not have any data on the constipation rate in HSCR children after pull-through who were going through or already had passed puberty. Therefore, it is interesting to conduct a cohort study to compare the constipation frequency between adolescent females and male HSCR patients.

The functional outcomes after pull-through were also associated with the peri-operative malnutrition [3]. Our study showed that there was no association between nutritional status of HSCR patients and their functional outcomes following pull-through. It should be noted that the small sample size, which was a weakness of our study, suggests that a larger sample of patients needs to be ascertained to clarify our findings.

Our study utilized the Krickenbeck classification to evaluate the functional outcomes following pull-through procedure according to previous studies [19–21]. However, it should be noted that the Krickenbeck classification was originally established for patients with anorectal malformation (ARM). There are different anatomies and associated anomalies between HSCR and ARM patients. The patients with HSCR possess normal anal canal and sphincter, and usually do not have any anomaly in the spinal cord and vertebrae. Therefore, our results should be interpreted with some caution given those differences. Caution should be also taken when generalizing about the findings since this is a mono-institutional study.

The algorithm has been proposed to improve the outcome of HSCR patients with soiling and constipation after pull-through [22]. The pediatric surgeon should begin with a detailed history and physical examination focused on the patient’s bowel habits and the method of the first pull-through, followed by several examinations, such as: contrast enema and rectal biopsy. Once the etiology of the symptoms after pull-through is established, it will be followed by specific treatment accordingly [22]. Moreover, our study also implies that the pediatric surgeon should monitor and evaluate closely the functional outcomes of their HSCR patients after pull-through to determine appropriate follow-up and management.

## Conclusions

The constipation rate is higher in patients who underwent Soave than Duhamel procedure, but the VBM and soiling frequencies are similar. The constipation risk following Soave pull-through might be increased by the female gender. Furthermore, a multicenter study with a larger sample of patients is necessary to clarify and confirm our findings.

## Abbreviations

HSCR: Hirschsprung disease
mo: months
TCA: Total colonic aganglionosis
VBM: Voluntary bowel movement
