# Correction to: Functional outcomes in Hirschsprung disease patients after transabdominal Soave and Duhamel procedures

**DOI:** 10.1186/s12876-018-0844-5

**Published:** 2018-07-09

**Authors:** Amira Widyasari, Winona Alda Pravitasari, Andi Dwihantoro, Gunadi

**Affiliations:** Pediatric Surgery Division, Department of Surgery, Faculty of Medicine, Universitas Gadjah Mada/Dr. Sardjito Hospital, Jl. Kesehatan No. 1, Yogyakarta, 55281 Indonesia

## Correction

After publication of the original article [1], the authors reported that the Acknowledgments section was incomplete. The full section is the following:
