# Correction to: Functional outcomes in Hirschsprung disease patients after transabdominal Soave and Duhamel procedures

**DOI:** 10.1186/s12876-021-01894-3

**Published:** 2021-09-30

**Authors:** Amira Widyasari, Winona Alda Pavitasari, Andi Dwihantoro, Gunadi

**Affiliations:** Pediatric Surgery Division, Department of Surgery, Faculty of Medicine, Universitas Gadjah Mada/Dr. Sardjito Hospital, Jl. Kesehatan No. 1, Yogyakarta, 55281 Indonesia

## Correction to: BMC Gastroenterology (2018) 18:56 https://doi.org/10.1186/s12876-018-0783-1

Following publication of the original article [1], the authors reported an error in the name of the second author; this should read Pavitasari.

The original article [1] has been corrected.
